# Body Size Variation in Italian Lesser Horseshoe Bats *Rhinolophus hipposideros* over 147 Years: Exploring the Effects of Climate Change, Urbanization and Geography

**DOI:** 10.3390/biology10010016

**Published:** 2020-12-30

**Authors:** Valeria B. Salinas-Ramos, Paolo Agnelli, Luciano Bosso, Leonardo Ancillotto, Víctor Sánchez-Cordero, Danilo Russo

**Affiliations:** 1 Wildlife Research Unit, Dipartimento di Agraria, Università degli Studi di Napoli Federico II, via Università, 100, 80055 Portici, Italy; valeria.salinasramos@unina.it (V.B.S.-R.); leonardo.ancillotto@unina.it (L.A.); 2 Sistema Museale dell’Università di Firenze, Museo di Storia Naturale, Sede di Zoologia La Specola, via Romana 17, 50125 Firenze, Italy; paolo.agnelli@unifi.it; 3 Laboratorio de Sistemas de Información Geográfica, Departamento de Zoología, Instituto de Biología, Universidad Nacional Autónoma de México, Av. Universidad, 04510 Ciudad de México, Mexico; victor@ib.unam.mx

**Keywords:** bat, Bergmann’s rule, climate change, land use change, light pollution, morphology, natural history collections

## Abstract

**Simple Summary:**

Animal body size varies in response to many environmental factors and may be influenced by climate change, food availability, habitat alterations or species interactions. Here, we use a specimen collection of Italian rhinolophid bats (*Rhinolophus hipposideros*) covering a long historical period (1869–2016) and looked at their body and skull size to see whether these changed over time and space. Although no temporal responses were recorded, which rules out an effect of climate change or urbanization, we found an increase in body size from south to north along the Italian territory which is best explained according to Bergmann’s rule. The latter postulates that larger individuals retain heat more effectively, so their presence in northern, colder climates is favoured, whereas, smaller ones dissipate heat more easily and are best adapted to cope with southern, warmer climates.

**Abstract:**

Body size in animals commonly shows geographic and temporal variations that may depend upon several environmental drivers, including climatic conditions, productivity, geography and species interactions. The topic of body size trends across time has gained momentum in recent years since this has been proposed as a third universal response to climate change along with changes in distribution and phenology. However, disentangling the genuine effects of climate change from those of other environmental factors is often far from trivial. In this study, we tested a set of hypotheses concerning body size variation across time and space in Italian populations of a rhinolophid bat, the lesser horseshoe bat *Rhinolophus hipposideros*. We examined forearm length (FAL) and cranial linear traits in a unique historical collection of this species covering years from 1869 to 2016, representing, to the best of our knowledge, the longest time series ever considered in a morphological assessment of a bat species. No temporal changes occurred, rejecting the hypotheses that body size varied in response to climate change or urbanization (light pollution). We found that FAL increased with latitude following a Bergmann’s rule trend, whereas the width of upper incisors, likely a diet-related trait, showed an opposite pattern which awaits explanation. We also confirmed that FAL is sexually dimorphic in this species and ruled out that insularity has any detectable effect on the linear traits we considered. This suggests that positive responses of body size to latitude do not mean per se that concurring temporal responses to climate change are also expected. Further investigations should explore the occurrence of these patterns over larger spatial scales and more species in order to detect the existence of general patterns across time and space.

## 1. Introduction

Climate change is one of the main threats to biodiversity and is well-known to exert several effects on the biota (e.g., [1]). In animals, reactions to climate change have been recorded, comprising three main categories, i.e., changes in geographic distribution [2], phenological modifications affecting migration [3], reproduction [4] or hibernation [5], and variation in body size [6]. A main hypothesis regarding the latter, at least for terrestrial endotherms, is that body size will decrease in response to a warming climate. The rationale behind this hypothesis is provided by the so-called Bergmann’s rule [7], according to which animal species, or, within species, individuals in warmer climates show a smaller body size than those living in colder climates. The rule rests on the fact that a smaller body size will correspond to a larger surface area/volume ratio, which translates into a more efficient heat dissipation—selected for under warmer conditions. On the other hand, a larger body size will be selected positively under colder conditions to retain body heat more effectively. When projected along a temporal gradient of increasing temperature such as that generated by climate change, this pattern would consist of a progressive reduction in body size, because smaller individuals will radiate heat more effectively, and would thus be better adapted to an increasing temperature scenario [8]. Yet, a smaller size implies greater vulnerability to dehydration and overheating in response to heat waves, so while a slow increase in temperature will select for smaller individuals, occasional thermal peaks would exert an opposite evolutionary force selecting for larger sizes. Studies have shown either pattern, as well as cases where no apparent body size trend was detected in response to climate change (reviewed in [8]). 

Insectivorous bats constitute an excellent study group to explore responses to climate change because key aspects of their biology are deeply influenced by ambient temperatures, such as hibernation [9], reproduction [10] and active foraging [11,12]. Moreover, bats possess a high body surface area relative to their volume, which exposes them to dehydration and heatwaves [13]. Either increases or decreases in bat body size are therefore valid hypotheses to explore as potential responses to climate change. Body size in Chinese *Rhinolophus pusillus*, for example, correlated negatively with the mean minimum temperature of the coldest month, supporting the fact that at least in that species body size is influenced by the need to preserve heat [14], although for bats no clear body size trend over time is known that might be associated with climate change. Prolonged pre-natal growth associated to higher temperatures under a climate change scenario might also lead to an increasing body size over time [15]. 

Italy is no exception to the global trend of warming temperature. Estimates of mean temperature increases in the country range between a 0.76 °C ± 0.19 °C in 1850–2005 [16] and an increase >1.1 °C of annual mean temperature in 1981–2010 over 1971–2000 [17], which sets the bases for investigating body size changes occurred over time in response to this alteration. However, drivers other than climate may cause changes in body size, confounding the pattern. For instance, spatial and temporal changes in body size may result from other factors, including variation in resource availability (named “resource rule” by [18]), competition [19] or prey-predator interactions [20]. Within this context, a special case of size variation over time in insectivorous bats is offered by the Kuhl’s pipistrelle *Pipistrellus kuhlii*, a thermophilous, ecologically flexible species that has experienced a four-fold increase in its European geographic distribution over the last decades best explained as a response to the warming climate [21]. Unlike many other light-averse bat species (e.g., [22]), *P. kuhlii* tolerates artificial illumination and, thus, frequently feasts on the swarms of positively phototactic insects attracted to streetlights [23,24,25]. In this species, body size showed no variation over ca. 130 years, but skull size increased after 1950, matching a boost in electric public illumination over the country [25]. Artificial illumination impairs antipredator evasive manoeuvres in tympanate moths triggered by bat echolocation [26], such moths, which would normally escape predation by *P. kuhlii*, can be caught by the bat near streetlamps. The increase in the bat cranial size over time might be a microevolutionary response, aimed at favouring capture of larger prey that has suddenly become available [25].

In our study, we examined *Rhinolophus hipposideros* from Italian populations collected in the period from 1869 to 2016. We provide a unique example of morphological analysis done on mammals over almost 150 years, and employ linear morphology to test alternative hypotheses about body size change over space and time in Italian populations of this bat species, as follows.

(1) Skull size and forearm length (hereafter FAL) of *R. hipposideros* will either decrease (to dissipate heat more effectively) or increase (to reduce the risk of dehydration) in response to a warming climate, so we predict an effect of the year of collection, which we adopt as a proxy for climate change as done in many other studies (see [27] for a review).
(2) *Rhinolophus hipposideros* skull size will not increase selectively over time, that is, there will be no influence of artificial illumination on this light-averse species, in contrast to light-exploiting species [25]. This rhinolophid is, in fact, an ideal control taxon in this context because it shows strong adverse reactions to artificial illumination [28].
(3) Finally, we formulate a set of geographic-framed hypotheses, i.e., that body size may respond to latitude, longitude, elevation or insularity. Specific predictions arising from such hypotheses are that: (a) Body size will increase at higher latitudes, in agreement with Bergmann’s rule, as seen in other bats [29]; and (b) bats from islands (Sardinia and Sicily) will be smaller (dwarfism) or larger (giantism) than their conspecifics from the mainland (insularity syndrome, [30]).

All models exploring the above-described hypotheses also included sex to control for its potential influence, since body size is sexually dimorphic in many bat species [31] including *R. hipposideros* [32], with females being significantly larger than males. 

## 2. Materials and Methods

### 2.1. Data Collection 

We measured FAL of 175 adult *R. hipposideros* (91 females, 84 males), for 73 of which skulls were sufficiently well-preserved to take reliable measurements. Six more skulls from specimens for which FAL was not available were included for analysis. All specimens are hosted at the ‘La Specola’ Zoological Museum of Florence University. Collection numbers are as follows: 3645, 3725, 3729–3732, 3734, 3736, 3773, 3788, 3805–3806, 3808, 3810–3811, 3852–3854, 3856–3857, 3890–3892, 3899, 3906, 3912–3915, 3919, 3923, 3925, 4481–4483, 4486–4490, 4500–4513, 4515–4525, 4527, 4529, 4534–4535, 4537–4552, 4556–4561, 4563–4568, 4570–4571, 4573, 4577–4579, 4581–4584, 4726–4733, 6711–6716, 7652–7655, 9047, 9052–9054, 9056, 9111–9115, 9117–9120, 9122–9125, 9128–9129, 9565, 9959, 9964–9965, 9974, 10501, 12631, 12641, 12761–12765, 13009, 13035, 13231, 13438, 13656, 14110–14111, 14114, 14315, 14323, 16735, 18906, 18913, 20613, 21314–21315, 22085. The time of collection spanned through three centuries: 99 specimens were from the 19th century (1869–1899), 73 from the 20th century (1904–1999), and two from the 21st century (2002, 2016). They originated from the entire Italian territory, including Sicily and Sardinia: Collection locations were comprised between latitudes 37.0° N and 41.1° N and altitudes of 0–2790 m a.s.l. Specimens were preserved in alcohol and all skulls extracted. The bat’s exact age could not be assessed due to the lack of ageing criteria, but dentition was consistent across specimens (V.B.S.-R., pers. obs.), so we ruled out that age could play a significant role in our results. 

We measured FAL on the right forelimb as a robust and reliable indicator of body size [33]. Moreover, since bat skull morphology may effectively unveil adaptive processes at the intraspecific level [34] we also characterised skull size using the same morphological parameters examined by [25]: greatest length of skull (GSL); mastoid breadth (MB); condylobasal length (CBL); cranial depth (CRD); width of upper incisors (I^2^I^2^); the length from the craniomandibular joint to the origin of the masseter muscle (A); and the length from the craniomandibular joint to the insertion of the masseter muscle, at the bottom of the angular process (B). V.S.R. measured such variables using an electronic digital calliper (0.01 mm precision) and expressed them in hundredths of a millimetre.

### 2.2. Data Analysis

**Figure 1:**
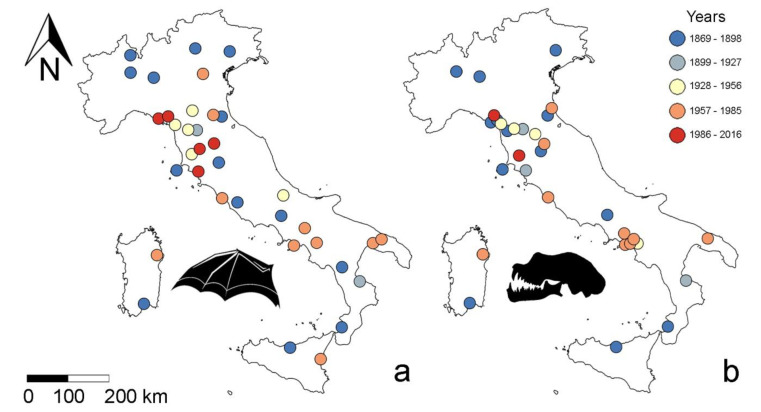
Geographic distribution of *Rhinolophus hipposideros* specimens from Italian populations considered for analysis after removing all spatially autocorrelated records. (**a**) Forearms: N = 36; (**b**) skulls: N = 31.

First, to avoid spatial autocorrelation, we screened records in ArcGis (v. 10.2.2) using the average nearest neighbour analysis to remove spatially correlated records and ensure independence (Supplementary Material Figures S1 and S2). This analysis calculates the mean distance (expressed as Euclidean distance) between presence records and compares it to the mean distance obtained for a random distribution [35,36,37]. This resulted in a forearm length sample size = 36 and skull sample size = 31 (Figure 1; Supplementary Material Tables S1 and S2). 

To explore the correlations between FAL and cranial variables, we used the whole sample and applied a Spearman’s rank correlation coefficient [38]. 

We assessed the influence of sex, year of collection, latitude, longitude (in degrees), elevation (in m) and insularity (island versus mainland specimen) on FAL employing a generalized linear model (GLM) analysis of covariance (ANCOVA). We carried out a Principal Component Analysis (PCA) on the seven cranial parameters to generate lower-dimensional data and test a subset of principal components that retained sufficient variation (e.g., [39]) against sex, year of collection, latitude, longitude (in degrees) and elevation (in m) in a general linear model multivariate analysis of variance (GLM MANOVA). In such analysis, the only categorical variable (sex) was entered as the main effect and the remaining, continuous variables as covariates. In this case, insularity was not tested for due to the small sample size of non-spatially autocorrelated insular records for which reliable cranial measurements were available (n = 3). We explored further the relationships between the principal components and the independent variables for which the GLM MANOVA led to significant results using regression analysis. We made sure that datasets conformed to univariate and multivariate test assumptions checking their structure with Ryan–Joiner and Mardia tests, and in case they did not, they were log-transformed to meet such assumptions. Significance was set at *p* < 0.05 and analyses were done with MINITAB 14. 

## 3. Results

FAL showed a moderately strong correlation only with CBL (r_s_ = 0.44, *p* < 0.001) and a weaker correlation with GSL (r_s_ = 0.29; *p* < 0.05), while correlations with the remaining five cranial variables were not significant. According to the GLM analysis, FAL was influenced significantly only by latitude and sex whereas the other factors had no effect (Table 1). 

**Figure 2:**
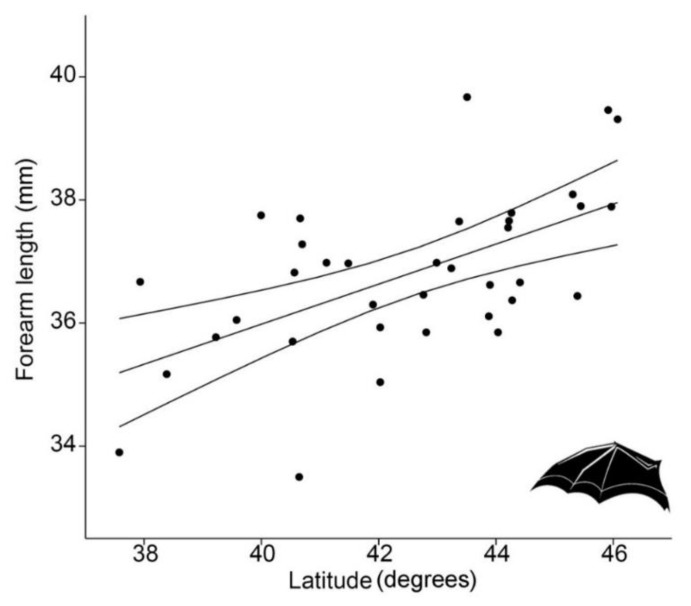
Linear regression (with 95% confidence intervals) between forearm length (FAL, in mm) of 36 *Rhinolophus hipposideros* from Italian populations and latitude (LAT, in degrees) (*p* < 0.0001, R^2^ = 33.1%).

FAL showed a strong latitudinal pattern, with larger individuals corresponding to higher latitudes (regression analysis, *p* < 0.0001, R^2^ = 33.1%) so it conformed to Bergmann’s rule (Figure 2). 

We also confirmed that FAL of females was greater than that of males both in the non-autocorrelated sample (females, 37.2 ± 1.27 mm, N = 15 vs. 36.5 mm ± 1.34 mm, N = 21, *p* < 0.001) and in the total sample (females, 37.3 ± 1.16 mm, N = 91 vs. males, 36.6 mm ± 1.20 mm, N = 84, *p* < 0.001).

The first four principal components arising from the PCA cumulatively expressed 0.35, 0.54, 0.68, and 0.80 of skull sample variance, respectively. Most variables provided a similar contribution to PC1, whereas PC2 was mostly influenced by B, A, MB the PC3 component was dominated by a negative loading provided by I^2^I^2^, and PC4 was negatively influenced by A and B (Table 2). 

We used such components to carry out the GLM MANOVA (Table 3), according to which only latitude influenced significantly the sample’s multivariate distribution (*p* < 0.05), whereas longitude, altitude, year of collection and sex had no effect. Separate regressions done using latitude as the independent variable and CBL, MB, B, GLS, CRD, I^2^I^2^ and A as dependent variables respectively showed that only PC3 increased significantly with latitude and was, thus, the factor that mostly influenced separation in the multivariate space (regression equation: PC3 = −30.3 + 8.08 Log Latitude; R^2^ = 18.0; F = 6,36, *p* < 0.002; non-significant results for PC1, PC2, PC4 vs. latitude regressions are not shown for brevity). Since PC3 was negatively dominated by I^2^I^2^ (Table 2), the latter parameter tends in fact to increase at lower latitudes. 

## 4. Discussion 

In this study, we found no temporal variation in forearm length and cranial size over ca. one century and a half in a rhinolophid bat species that is sensitive to human alteration of landscapes, including light pollution. Specifically, neither FAL nor cranial dimensions varied temporally, ruling out the existence of responses to climate change and indirectly confirming the hypothesis based on [25] that no illumination-driven cranial size variation should occur in a light-averse bat species. 

The lack of temporal trends in body size also rejects alternative interpretations, such as, for instance, the possible occurrence of any response to changes in land use, and consequently, productivity, according to the “resource rule” pattern [18]. *Rhinolophus hipposideros* mostly prey on dipterans and moths, which in many cases have declined due to land use change [40], so factors such as agricultural intensification, urban expansion and forest reduction and fragmentation have likely contributed to a reduction in food availability for this bat [41]. As a pest suppressor [42], *R. hipposideros* is also exposed to the direct and indirect effects of pesticides [43]. Although a positive population trend was recorded in 1993–2011 across European hibernacula [44], in Italy *R. hipposideros* is still at risk and classified as endangered in the Italian Red List of Vertebrates [45]. Clearly, the adverse effects of land use change do not necessarily reveal themselves as a modification in linear traits such as FAL or cranial variables, which tend to be less variable than body mass [27]. Reductions in habitat or food availability may affect aspects other than body size at the individual level, such as mortality and fitness [46]. 

The authors in [15] compared specimens collected in the ‘60s versus others collected in 2017 of a frugivorous (*Rousettus leschenaultia*) and an insectivorous (*Hipposideros armiger*) bat species, respectively, and found that the former showed changes in diet-related cranial traits, while the latter increased both FAL and cranial variables, as well as carbon and nitrogen isotope contents. In both cases, this was interpreted as a response to land use change, and, in turn, food availability rather than climate change.

An alternative hypothesis proposed for larger skull sizes and thus, larger brain volumes observed in urbanized landscapes, is that urbanization would select for individuals possessing higher cognitive skills, capable of coping with the unpredictable nature of urban environment [47,48]. From this viewpoint too, we did not expect any variation in cranial capacity over time (i.e., over a temporal gradient of urbanization): *R. hipposideros* may roost in buildings, especially in marginal urban settlements, but it is not an urban exploiter and its main foraging habitat is the forest [49]. Research has shown mixed results, however (e.g., [50]): For instance, a study on the effects of urbanization on birds rejected the hypothesis that large brains favour colonization of urban habitat, but proposed a relationship between skewness in brain size and this capacity [51]. 

We are aware that the lack of change in body size may result from limited sample size rather than representing a genuine pattern [27], and that we deliberately reduced our sample size to avoid spatial autocorrelation. Yet, this (often neglected) choice was important to avoid overrepresentation of areas for which more material was available. Moreover, the sample size we used could still successfully detect body patterns in space, which makes us confident of the reliability of our results. 

The evidence we provide that body size in *R. hipposideros* varies in response to latitude is in agreement with Bergmann’s rule [52], since we found a clear positive relationship between latitude and FAL. Whether this represents a reaction to thermoregulatory requirements under different climatic conditions rather than to latitude-dependent productivity is an open question [27]. Noticeably, FAL responded positively to latitude but did not change over time, suggesting that the existence of Bergmann’s rule trends does not imply that temporal responses to the warming climate are also expected. In *P. kuhlii*, no latitudinal effect on FAL was recorded, showing that such patterns are species-specific [25]. This might be an effect of a deeper genetic structuring of *R. hipposideros* compared to *P. kuhlii*, which is a generalist, common bat species not affected by fragmentation unlike the former. 

We found that neither longitude nor elevation influenced body size (FAL and cranial dimension), and rejected the hypothesis that either dwarfism or gigantism occurred, although we caution that insular sample size of FAL was limited. However, our results agree with [32], which, based on current specimens, also found no FAL difference between Sardinian and mainland populations of this species, as well as of *R. euryale*.

Cranial measurements lacked any temporal or spatial patterns with the only exclusion of PC3, which basically expressed an inverse relationship between I^2^I^2^ and latitude. This observation, along with the significant correlations found between FAL and two cranial length parameters (CBL and GSL) show that in bats as in other mammals [53], skull length is correlated with body size. In bats, other important ecomorphological constraints act over cranial design, such as echolocation, food acquisition and processing [54], which may lead to departure from size-scaling relationships. The functional significance of the inverse relationship between I^2^I^2^ and latitude is unknown and might simply reflect a causal relationship between the former parameter and a diet-related variable that was neglected in our analysis, such as food type availability at different latitudes [15]. 

## 5. Conclusions

From a methodological viewpoint, our work confirms the importance of examining historical material preserved in natural history collections [55], in order to answer ecological and evolutionary questions regarding patterns in space and time. However, this approach is constrained by the limited availability of comprehensive temporal series of samples. We also found forearm length to be more sensitive to geographical factors than are cranial measurements. This finding reinforces the importance of forearm length in bat research. This variable can be measured readily and non-invasively, and large datasets may be gathered to explore spatial and temporal patterns at different scales. Our work contributes towards a better understanding of the environmental correlates of bat body size, and shows that generalizations are difficult because responses to climate and land-use change differ across species. Future research should focus on exploring spatial and temporal patterns across a larger geographic scale—ideally in Europe—including more bat species, and relating taxonomical and functional diversity to spatiotemporal climate and land-use variation.

## Figures and Tables

**Table 1:** General linear model (GLM) analysis of covariance (ANCOVA) of forearm length in 36 specimens of *Rhinolophus hipposideros* from Italian populations collected between 1869 and 2016. Data that did not meet test assumptions were log-transformed for analysis.

Source	d.f.	F	*p*
Latitude (decimal degrees)	1	4.27	<0.05
Longitude (decimal degrees)	1	0.02	n.s.
Altitude (m a.s.l.)	1	0.98	n.s.
Year of collection	1	0.29	n.s.
Sex	1	5.95	<0.05
Mainland vs. Island	1	0.08	n.s.
Error	29		
Total	35		

**Table 2:** Loadings for the first four components from a principal component analysis of seven cranial variables of *Rhinolophus hipposideros* (n = 31) from Italian populations collected between 1869 and 2002.

Cranial Variable	PC1	PC2	PC3	PC4
Greatest length of skull (GSL)	−0.536	−0.030	0.143	−0.141
Condylobasal length (CBL)	−0.490	−0.070	0.178	0.174
Mastoid breadth (MB)	−0.398	−0.477	0.116	−0.095
Cranial depth (CRD)	−0.488	0.188	−0.089	0.295
Width of upper incisors (I2I2)	−0.227	0.168	−0.917	−0.094
Length from the craniomandibular joint to the origin of the masseter muscle (A)	−0.150	0.494	0.200	−0.793
Length from the craniomandibular joint to the insertion of the masseter muscle (B)	0.052	−0.678	−0.213	−0.465

**Table 3:** Results of general linear model multivariate analysis of variance (GLM MANOVA) on the first five Principal Components obtained from a PCA done on cranial measurements of *Rhinolophus hipposideros* from Italian populations (n = 31) collected between 1869–2002. Sex (a categorical variable) was entered as the main factor, all other (continuous) variables as covariates. Data that did not meet test assumption were log-transformed for analysis.

Variable	Wilk’s Statistic	d.f.	F	*p*
Latitude (decimal degrees)	0.646	4, 21	2.873	<0.05
Longitude (decimal degrees)	0.778	4, 21	1.497	n.s.
Altitude (m a.s.l.)	0.823	4, 21	1.127	n.s.
Year of collection	0.785	4, 21	1.438	n.s.
Sex	0.888	8, 42	0.319	n.s.

## Data Availability

The data presented in this study are available in the Supplementary Material annexed to this article.

## Supplementary Materials

The following items are available online at https://www.mdpi.com/2079-7737/10/1/16/s1, Figure S1: Average Nearest Neighbor summary obtained from spatial autocorrelation analysis of forearm length of Italian *Rhinolophus hipposideros*, Figure S2: Average Nearest Neighbor summary obtained from spatial autocorrelation analysis of skull variables of Italian *Rhinolophus hipposideros*, Table S1: Main data used for *Rhinolophus hipposideros* forearm length analysis selected according to spatial autocorrelation analysis. All specimens are hosted at the ‘La Specola’ Zoological Museum of Florence University. Values are expressed in mm, Table S2: Main data used for *Rhinolophus hipposideros* skull analysis selected according to spatial autocorrelation analysis. All specimens are hosted at the ‘La Specola’ Zoological Museum of Florence University. CRD = Cranial depth; GSL = Greatest length of skull; CBL = Condylobasal length; MB = Mastoid breadth; A = Length from the craniomandibular joint to the origin of the masseter muscle; B = Length from the craniomandibular joint to the insertion of the masseter muscle. Values are expressed in mm.
