# Supplementary material for: Body Size Variation in Italian Lesser Horseshoe Bats Rhinolophus hipposideros over 147 Years: Exploring the Effects of Climate Change, Urbanization and Geography
Source: Biology (Basel). 2020 Dec 30;10(1):16. doi: 10.3390/biology10010016 (PMC7824098; doi:10.3390/biology10010016)
Supplement: Supplementary file 1 [file biology-10-00016-s001.pdf]

Average Nearest Neighbor Summary

Nearest Neighbor Ratio: 0,875924
z-score: -1,424195
p-value: 0,154390

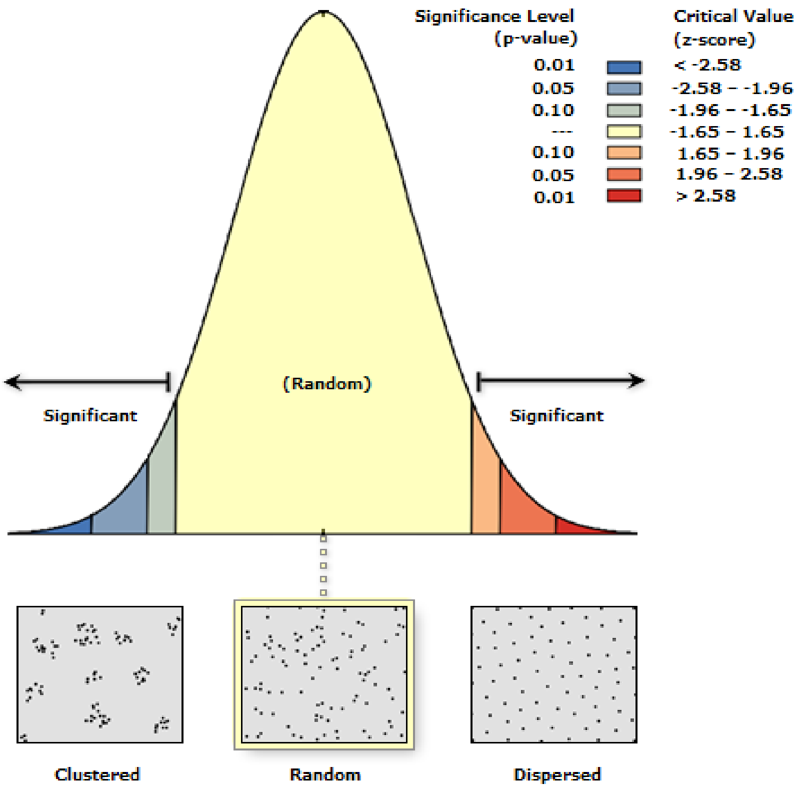

Given the z-score of -1.42419481441, the pattern does not appear to be significantly different than random.

**Observed Mean Distance:**	60081,0228 Meters
**Expected Mean Distance:**	68591,5817 Meters
**Nearest Neighbor Ratio:**	0,875924
**z-score:**	-1,424195
**p-value:**	0,154390

Figure S1. Average Nearest Neighbor summary obtained from the spatial autocorrelation analysis of the forearm data of *Rhinolophus hipposideros* in Italy.

Average Nearest Neighbor Summary

Nearest Neighbor Ratio: 0,866383
z-score: -1,423220
p-value: 0,154672

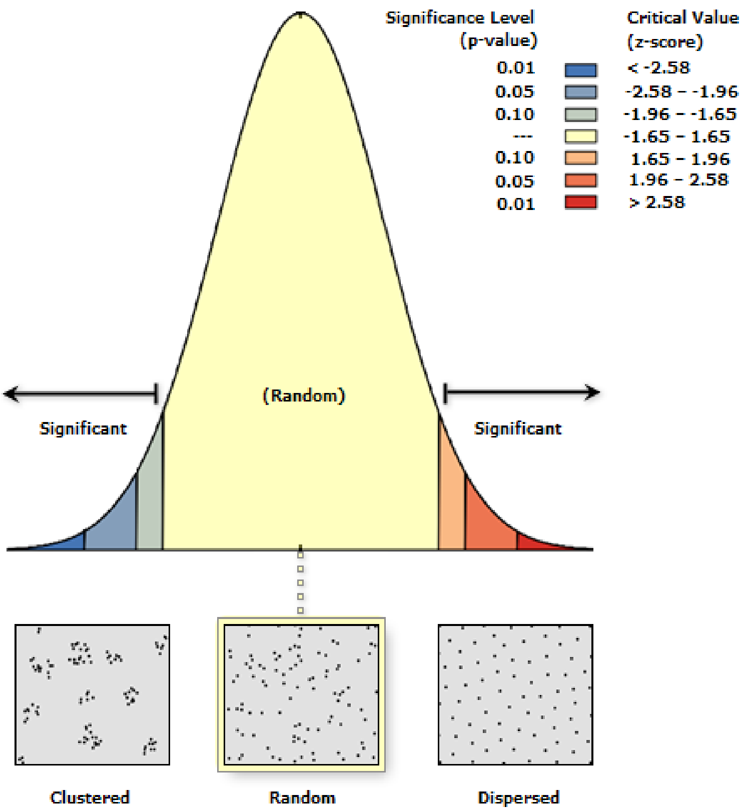

Given the z-score of -1.42322031688, the pattern does not appear to be significantly different than random.

**Observed Mean Distance:**	62024,3211 Meters
**Expected Mean Distance:**	71589,9229 Meters
**Nearest Neighbor Ratio:**	0,866383
**z-score:**	-1,423220
**p-value:**	0,154672

Figure S2. Average Nearest Neighbor summary obtained from the spatial autocorrelation analysis of the skull data of *Rhinolophus hipposideros* in Italy.

Table S1: Main data used for *Rhinolophus hipposideros* forearm length analysis selected according to spatial autocorrelation analysis. All specimens are hosted at the 'La Specola' Zoological Museum of Florence University. Values are expressed in mm.

Year	Id record	Sex	Region	Province	Forearm length
1869	4549	F	Sicilia	Palermo	36.67
1873	4581	F	Sardegna	Cagliari	35.77
1874	4511	F	Piemonte	Novara	37.90
1876	4516	M	Emilia Romagna	Forlì Cesena e Rimini	37.66
1877	4563	M	Toscana	Siena	36.98
1877	4527	M	Toscana	Livorno	35.85
1877	4560	F	Calabria	Reggio Calabria	35.17
1878	13231	F	Basilicata	Potenza	37.75
1878	4538	M	Friuli	Pordenone	37.89
1879	4481	F	Molise	Isernia	36.97
1892	9056	M	Lazio	Roma	36.30
1895	9120	M	Lombardia	Como	39.46
1896	9122	F	Lombardia	Lodi	38.09
1898	9047	F	Trentino Alto Adige	Trento	39.31
1899	9053	M	Toscana	Prato	36.11
1904	9123	F	Calabria	Cosenza	36.05
1949	3906	F	Abruzzo	Chieti	35.93
1951	3892	M	Toscana	Pisa	36.89
1953	3853	M	Toscana	Pistoia	36.62
1954	3805	M	Toscana	Lucca	35.85
1955	3773	M	Emilia Romagna	Modena	36.66
1957	14110	M	Lazio	Roma	35.04
1959	3899	F	Puglia	Taranto	36.82
1962	3734	M	Puglia	Brindisi	33.50
1965	3788	M	Veneto	Vicenza	36.44
1965	3912	M	Campania	Napoli	37.70
1965	3913	M	Campania	Salerno	37.28
1965	3915	M	Campania		36.98
1974	9565	M	Sicilia	Catania	33.90
1982	13009	M	Sardegna	Nuoro	35.70
1984	12765	M	Emilia Romagna	Ravenna	36.37
1992	13656	F	Liguria	La Spezia	37.55
1992	13438	F	Toscana	Grosseto	36.46
1997	16735	F	Toscana	Arezzo	39.67
2002	20613	F	Toscana	Massa Carrara	37.79
2016	22085	M	Toscana	Siena	37.65

Table S2: Main data used for *Rhinolophus hipposideros* skull analysis selected according to spatial autocorrelation analysis. All specimens are hosted at the 'La Specola' Zoological Museum of Florence University. CRD = Cranial depth; GSL = Greatest length of skull; CBL = Condylobasal length; MB = Mastoid breadth; A = Length from the craniomandibular joint to the origin of the masseter muscle; B = Length from the craniomandibular joint to the insertion of the masseter muscle. Values are expressed in mm.

Year	Id record	Sex	Region	Province	CRD	GSL	CBL	MB	A	I2I2	B
1869	4551	F	Sicilia	Palermo	6.03	14.28	13.21	7.19	3.14	1.52	1.96
1873	4579	M	Sardegna	Cagliari	5.87	14.57	13.06	7.14	3.68	1.42	2.06
1874	4511	F	Piemonte	Novara	5.92	15.00	13.54	7.52	4.00	1.35	2.33
1876	4726	M	Liguria	La Spezia	6.02	15.23	13.42	7.32	3.54	1.31	2.41
1876	4515	M	Emilia Romagna	Forlì Cesena e Rimini	6.06	15.5	14.03	7.23	3.62	1.13	1.96
1876	4573	F	Toscana	Massa Carrara	5.89	14.85	13.79	7.40	3.78	1.11	1.94
1877	4486	M	Toscana	Arezzo	6.04	15.27	13.81	7.30	3.90	1.29	1.98
1877	4534	F	Toscana	Livorno	5.70	14.93	13.08	7.10	3.87	1.24	1.89
1877	4561	F	Calabria	Reggio Calabria	6.21	15.72	13.86	7.15	4.07	1.88	1.88
1877	4571	M	Toscana	Lucca e Massa Carrara	5.76	14.85	13.52	7.22	4.39	1.24	2.06
1878	4537	M	Friuli	Pordenone	6.00	15.40	13.64	7.51	3.50	1.50	2.06
1879	4481	F	Molise	Isernia	5.85	14.77	13.49	7.28	3.34	1.15	2.33
1896	9122	F	Lombardia	Lodi	5.74	15.04	13.61	7.38	3.80	1.35	1.90
1904	9124	F	Calabria	Cosenza	5.73	15.28	13.29	7.43	3.42	1.22	2.39
1905	9111	M	Toscana	Grosseto	5.66	14.85	13.00	7.14	3.43	1.14	2.40
1906	10501	F	Toscana	Prato	6.03	14.89	13.46	7.12	3.77	1.25	2.08
1953	3852	F	Toscana	Pistoia	5.99	15.61	14.06	7.40	4.07	1.22	2.07
1954	3805	M	Toscana	Lucca	5.97	14.75	13.41	7.20	3.64	1.35	1.76
1954	9964	M	Campania	Salerno	6.01	15.11	13.72	7.35	3.67	1.40	2.41
1955	3731	M	Toscana	Firenze	5.84	15.11	13.38	7.19	4.43	1.17	2.08
1957	14114	M	Lazio	Roma	6.96	16.04	13.58	7.41	4.21	1.35	1.95
1962	3734	M	Puglia	Brindisi	5.99	14.97	13.46	7.31	4.55	1.53	2.11
1963	9380	M	Toscana	Arezzo	5.80	14.78	13.48	7.38	3.83	1.24	2.04
1965	9974	F	Campania	Avellino	5.94	14.65	13.96	7.30	4.10	1.38	1.93
1970	9959	M	Campania	Salerno	6.11	14.90	13.54	7.27	3.46	1.16	1.88
1982	13009	M	Sardegna	Nuoro	5.83	14.46	13.38	7.34	3.90	1.33	1.88
1983	14122	0	Campania	Salerno	6.06	15.62	13.5	7.49	3.75	1.37	2.13
1984	12762	M	Emilia Romagna	Ravenna	5.86	14.82	13.63	7.34	3.73	1.58	2.57
1985	0	M	Campania	Salerno	6.00	15.01	13.37	7.21	3.89	1.10	2.10
1999	18913	F	Toscana	Siena	5.68	14.4	12.78	7.05	4.05	1.23	2.00
2002	20613	F	Toscana	Massa Carrara	5.80	15.20	13.14	7.25	4.51	1.31	2.09
